# The Role of Peptides in Asthma–Obesity Phenotype

**DOI:** 10.3390/ijms25063213

**Published:** 2024-03-12

**Authors:** Ewelina Russjan

**Affiliations:** Department of Respiration Physiology, Mossakowski Medical Research Institute, Polish Academy of Sciences, Pawińskiego 5 St., 02-106 Warsaw, Poland; erussjan@imdik.pan.pl

**Keywords:** asthma, obesity, regulatory peptides, inflammation

## Abstract

The co-occurrence of asthma and obesity is becoming an increasingly common health problem. It became clear that both diseases are closely related, since overweight/obesity are associated with an increased risk of asthma development, and more than half of the subjects with severe or difficult-to-treat asthma are obese. Currently, there are no specific guidelines for the treatment of this group of patients. The mechanisms involved in the asthma–obesity phenotype include low-grade chronic inflammation and changes in pulmonary physiology. However, genetic predispositions, gender differences, comorbid conditions, and gut microbiota also seem to be important. Regulatory peptides affect many processes related to the functioning of the respiratory tract and adipose tissue. Adipokines such as leptin, adiponectin, resistin, and the less studied omentin, chemerin, and visfatin, as well as the gastrointestinal hormones ghrelin, cholecystokinin, glucagon-like peptide-1, and neuropeptides, including substance P or neuropeptide Y, can play a significant role in asthma with obesity. The aim of this article is to provide a concise review of the contribution of particular peptides in inflammatory reactions, obesity, asthma, and a combination of both diseases, as well as emphasize their potential role in the effective treatment of the asthma–obesity phenotype in the future.

## 1. Introduction

Bronchial asthma and obesity have become a major health problem, affecting people throughout the world, which is reflected in the steady increase in the incidence of these conditions. Asthma is a chronic disease characterized by inflammation and variable obstruction of the airways, resulting in characteristic symptoms such as wheezing, chest tightness, shortness of breath, and cough [1]. The prevalence of asthma is estimated to be 358 million people worldwide, making it the most common respiratory disease [2]. At the same time, more than 1.9 billion adults are overweight and more than 650 million of these are obese, representing 39% and 13% of the whole population, respectively [3]. Obesity is classically defined as a body mass index (BMI) ≥ 30 kg/m^2^. However, the BMI parameter, although the simplest, may not be the most appropriate to assess the effect of adiposity on other health conditions. It becomes clear that not only total body mass but also fat distribution plays an important role in this relationship. In fact, fat accumulated in the abdominal subcutaneous and visceral region, which especially increases the risk of obesity complications, could have different metabolic activity, compared to peripheral adipose tissue [4]. To determine the level of abdominal obesity, other indices, such as waist circumference (WC), waist–hip ratio (WHR), or waist-to-height ratio (WHtR), can be used [5]. Interestingly, there is evidence that measurement of both abdominal obesity (WC) and general obesity (BMI) appears to be more favorable than using each of these parameters alone to assess the risk of asthma in the female population [6].

The link between asthma and obesity was described almost 40 years ago. The first report on this topic comes from 1986 when Seidell et al. demonstrated an association between asthma/bronchitis and severe obesity in women [7]. Subsequently, in 1999, Camargo et al. conducted a prospective cohort study, investigating nearly 86,000 female participants (the Nurses’ Health Study II) and showed that BMI is strongly and positively related to the risk of adult-onset asthma [8]. Similar observations have been made in a longitudinal study of 135,000 Norwegian men and women, pointing to an increased risk of adult asthma in overweight and obese individuals [9]. The meta-analysis of 2007, including the studies mentioned above and several others, indicated that there is a positive correlation between BMI and asthma morbidity, and sex has no impact on this relationship [10]. At present, the medical guidelines organization Global Initiative for Asthma (GINA) identifies asthma with obesity as one of the most common clinical phenotypes of the disease [1]. In fact, almost 60% of patients with severe or difficult-to-treat asthma suffer from obesity [11], and overweight/obesity are associated with a 1.5- to 2.5-fold increase in the risk of asthma incidence [12].

This article first explains the differences between two distinct phenotypes of obese asthma, gives insights into the mechanisms responsible for the asthma–obesity interactions and describes the effectiveness of available therapy. Later on, as the main purpose of the review, characteristics of regulatory peptides, including their role in inflammation, obesity, or asthma, and a summary of the current state of knowledge about the contribution of selected peptides in the asthma–obesity phenotype, together with possible therapeutic use in the future, are provided.

## 2. Asthma–Obesity Phenotype

The asthma–obesity causal relationship is complex and appears to be bidirectional. While obesity is well described as a risk factor for bronchial asthma [13], the question is whether asthma contributes to obesity development. A prospective study of the school-aged population demonstrated that children with a diagnosis of asthma might be at increased risk of becoming obese, and it can be prevented by using asthma rescue medication [14]. Moreover, a multicenter longitudinal study has shown that early-onset asthma and wheezing may lead to a higher risk of obesity in later childhood [15]. With regard to these observations, obese asthma cannot be treated as a homogeneous entity. The current evidence suggests that there are at least two distinct phenotypes of disease. The first one is referred to as early-onset asthma complicated by obesity with a similar prevalence in both sexes and characteristic features of the allergic process, e.g., an increased level of serum IgE. In this type, usually atopic asthma is associated with more severe airway obstruction as well as more pronounced bronchial hyperresponsiveness in comparison to nonobese asthmatic patients [16,17]. Due to the fact that early-onset asthma begins in childhood, the impact of maternal weight on the incidence of asthma in the offspring seems to be significant. The results of a meta-analysis that includes fourteen studies indicate that maternal obesity or increased weight gain during pregnancy represent risk factors for asthma or wheezing in children [18]. Moreover, the latest research shows that maternal history of asthma and pregnancy hypertension increase the chances of asthma and overweight/obesity in children at 6 and 7 years of age [19].

The second phenotype is late-onset asthma developing as a consequence of obesity. This variant most often affects women and is characterized by poorly controlled asthma, a more severe course of the disease, and resistance to corticosteroid therapy. At the cellular level, asthma caused by obesity has a nonatopic nature, with a limited level of serum IgE and predominantly neutrophil infiltration into the airway [20]. Some authors go a step further and suggest the existence of a distinct neutrophilic asthma–obesity phenotype among patients with late-onset asthma [21]. Interestingly, in early-onset asthma, weight loss does not cause remission of the disease, although its symptoms might decrease. On the contrary, in patients with a late-onset form, the disease can regress after weight reduction, clearly indicating that their asthma is the outcome of obesity [22]. Additionally, the factor that complicates our understanding of the co-occurrence of asthma and obesity is that obesity can cause symptoms that mimic asthma, as a result of typical changes in lung function during obesity or obesity-related comorbidities [23]. However, the longitudinal study has demonstrated that overdiagnosis of asthma does not occur more frequently in obese compared to nonobese patients [24].

## 3. Interactions between Asthma and Obesity

There are many mechanisms that could explain the relationship between asthma and obesity, and the most obvious is the effect of excess abdominal and thoracic adipose tissue on pulmonary physiology. Obesity may cause alterations in the airways that include increased resistance and breathing work, inefficiency of respiratory muscle, decreased respiratory compliance, or modification in gas exchange. The abnormalities observed in obese individuals, the reduction in expiratory reserve volume (ERV), and a correlated decrease in functional residual capacity (FRC) are proportional to increasing BMI [25]. It was demonstrated that at a BMI = 30 kg/m^2^, FRC and ERV parameters account for 75% and 47% of the values obtained for patients with a BMI = 20 kg/m^2^ [26]. Morbid obesity may be associated with lower total lung capacity (TLC), however, the changes are relatively minor [26,27]. Some studies also show that obesity is related to reduced forced expiratory volume in 1s (FEV_1_) and forced vital capacity (FVC) [28]; in others, the differences between lean and obese asthmatics were only slight [29]. Both parameters are usually reduced to a similar degree, and consequently, the FEV_1_–FVC ratio remains unchanged [30].

The impact of obesity on airway hyperresponsiveness (AHR) is the subject of debate, and the findings are not conclusive. In a large multicenter study including more than 11,000 participants, Chinn has shown that a higher BMI was correlated with a more pronounced AHR to methacholine. The effect was rather weak, and significant differences were observed only in men and not in women [31]. Litonjua, in turn, has demonstrated that, in the population of adult men, risk factors for hyperresponsiveness development are both low and high BMI values [32]. However, a large body of evidence has documented that there is no association between obesity and increased AHR. The lack of such a relationship, in either men or women, has been confirmed by Hancox et al. in the cohort study of around 1000 subjects [33]. Similar results were also observed among adolescents aged 12–18 years in response to a provocative dose of methacholine [34] and in adult patients exposed to histamine inhalation [35].

In the full understanding of the asthma–obesity correlation, not less important is the fact that adipose tissue is a metabolically active organ, producing a variety of mediators, which leads to chronic, low-grade systemic inflammation. It has been postulated that this inflammatory state can modulate airway inflammation and contribute to asthma development in obese individuals [36]. Cells of adipose tissue are a source of molecules, collectively referred to as adipokines, including interleukin 6 (IL-6), tumor necrosis factor alpha (TNF-α), transforming growth factor beta (TGF-β), C-reactive protein (CRP), eotaxin, leptin, and adiponectin [37]. IL-6, as a pleiotropic cytokine, is believed to play an important role in the pathogenesis of both diseases. It was demonstrated that obese patients with asthma had elevated levels of IL-6 in serum in comparison with overweight and nonobese subjects, and this was associated with asthma severity [38]. Similar results were obtained in a morbidly obese population, where the group of asthmatics had an increased level of IL-6 and the number of neutrophils in peripheral blood compared to the nonasthmatic morbidly obese [39]. Furthermore, a high plasma concentration in asthma was correlated not only with a higher body mass index but also with a higher incidence of diabetes and hypertension, impaired lung function, and increased risk of asthma exacerbation [40]. In children, a relationship was found between serum IL-6 and BMI percentiles, however, no correlation between IL-6 and asthma severity was observed [41]. TNF-α, in turn, is a proinflammatory cytokine produced by macrophages, mast cells, and adipocytes, as well as fibroblasts, smooth muscle cells, and epithelial cells [36]. Canöz et al. have shown that serum levels of TNF-α, IL-6, and leptin were significantly higher in obese asthmatics than in normal-weight asthma patients, and it was correlated with BMI, WC, and WHR parameters [42]. It has been shown that TNF-α can elevate the serum level of leptin in humans [43], and, conversely, leptin can affect the production of TNF-α and IL-6 within adipose tissue [44]. The detailed role of leptin and other adipokines will be described later.

The results of several studies support the hypothesis that the asthma–obesity phenotype is characterized by a neutrophilic, rather than eosinophilic, pattern of airway inflammation. Von Mutius has shown no correlation between body mass and atopy or eosinophils in serum, indicating that other mechanisms than allergic inflammatory reaction may be involved in obesity–asthma co-occurrence in children [45]. Similar observations have been made in adult studies, demonstrating an association between central obesity and nonatopic, but not atopic asthma in men and women [46], as well as a higher risk of obese asthma in nonallergic rather than allergic subjects [47]. In the study by Scott et al., the sputum neutrophil percentage was increased in obese asthmatic subjects compared to lean asthmatics and obese control patients. In addition, the percentage of sputum eosinophils was higher in the asthma, but not in the obesity group. However, in the asthma group, the percentage of neutrophils in the sputum was positively correlated with BMI only in women, not in men [48]. These results are confirmed by another report in which obese asthma was associated with a significantly elevated number of neutrophils in the blood and are in agreement with previous findings; this relationship was observed only in the female population [49]. It is worth noting that neutrophilic inflammation may be related to an increased level of IL-17A, which acting as a chemoattractant, recruits neutrophils to the airways [50]. In humans, obese asthma was associated with an increased number of neutrophils (but not eosinophils) and a high level of IL-17A mRNA in the sputum, which showed a positive correlation with body mass index [51]. In the animal model, obese mice on a high-fat diet exhibited a markedly elevated concentration of IL-17A in the lung and developed innate hyperresponsiveness. The presence of IL-17A turned out to be necessary for obesity-induced AHR, as it was not seen in the IL-17A-deficient animals [52]. Furthermore, obesity-related asthma is characterized by increased oxidative stress, pronounced mostly by elevated levels of free radicals and decreased activity of antioxidant systems [53]. Komakula et al. have shown that body mass index is correlated with elevated exhaled 8-isoprostanes, a biomarker of airway oxidative stress in asthmatics [54]. However, systemic oxidative stress assessed by plasma concentrations of F2-isoprostane was not independently related to asthma and cannot be responsible for the asthma–obesity relationship [53].

Although substantial changes in lung physiology and chronic inflammatory state appear to be crucial factors explaining the connection between asthma and obesity, other aspects, including genetic component, gender, and the role of sex hormones, comorbidities or gut microbiota may also be important. Indeed, a study of 1001 monozygotic and 383 dizygotic same-sex twin pairs revealed a significant correlation between asthma and BMI with strong heritability at the level of 77% for obesity and 53% for asthma, pointing to the additive genetic impacts on each disease [55]. Moreover, it has been demonstrated that some regions of the human genome, such as chromosomes 5q, 6p, 11q, and 12q, are associated with both asthma and obesity, containing genes encoding TNF-α, glucocorticoid receptor, or β2-adrenoceptor [56]. Another important issue, in addition to genetic determinants, seems to be gender differences, and although many studies indicate a similar prevalence of obese asthma in both sexes [9,10], some reports suggest that this phenotype is more common in the female population [33,57]. It may be the result of sex hormones’ activity since it has been shown that asthma severity increases with body weight in women but not in men. And, this correlation was more pronounced in women with early menarche [58]. Similarly, Castro-Rodrguez et al. have found that becoming overweight/obese during school years (between ages six and eleven) in girls was associated with a seven-fold increased risk of new asthma symptoms. The most obvious correlation was described among women with puberty onset before the age of eleven [59]. However, a strong impact on asthma with obesity may also have comorbid conditions (including obstructive sleep apnea (OSA), gastroesophageal reflux disease (GERD), or metabolic syndrome), which can affect asthma manifestations [12]. OSA and habitual snoring, classified as sleep-disordered breathing (SDB), are associated with asthma and wheezing symptoms. The relationship between wheezing and obesity might be to some extent explained by factors correlated with SDB [60]. Moreover, subjects with GERD are more likely to develop concurrent asthma in comparison with subjects without GERD [61], and GERD is more often observed in obese asthmatic patients than in patients with normal weight [62,63]. However, other authors reported that the effect of OSA on asthma control is not dependent on obesity [64] and that GERD is not responsible for more severe asthma symptoms in obese individuals [65]. The presence of metabolic syndrome, which involves central obesity, type-2 diabetes, hypertension, and dyslipidemia, may also be of importance. In fact, the association between metabolic syndrome and lung function impairment [66], as well as between metabolic syndrome and asthma-like symptoms, including wheezing, postexercise dyspnea, and dyspnea at rest, has been demonstrated [67].

**Figure 1:**
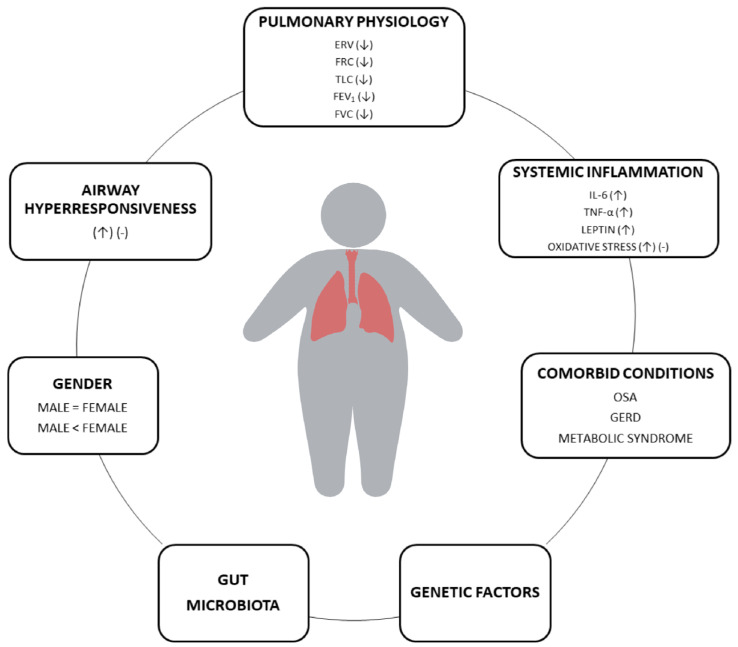
The mechanisms involved in asthma–obesity phenotype. (↑) increase, (↓) decrease, (-) no difference.

Another factor that may play an important role in the interaction between asthma and obesity is the gut microbiota. Several animal studies indicate that diet can significantly affect microbial composition [68,69], and obesity is characterized by decreased bacterial diversity with increasing *Firmicutes* abundance and a decline in the phylum *Bacteroidetes* [70,71,72]. A recent study indicates that the gut microbial community of pregnant women exerts a strong influence on the composition of the umbilical cord plasma, including levels of cytokines, immunoglobulins, and adipokines [73]. Modification of intestinal microbiota has also been demonstrated in adult asthmatic patients compared to healthy individuals. It can lead to altered production of short-chain fatty acids, which regulate Th cells’ activity and are involved in inflammatory reactions [74]. In addition, an early reduction of microbial diversity in the gut can contribute to asthma and allergy development as a result of Th1–Th2 response imbalance [75]. In the case of asthma with obesity, Michalovich et al. have shown that the co-occurrence of both diseases exerts an additive effect in exaggerating changes in the microbial composition. Moreover, the severity of asthma was negatively related to fecal levels of gut bacteria *Akkermansia muciniphila* [76]. The most important mechanisms involved in the asthma–obesity phenotype are presented in Figure 1.

## 4. Challenges in Therapy of Asthma with Obesity

Currently, there are no specific recommendations for the treatment of obese asthmatics. However, it is assumed that weight loss may have an advantageous effect on asthma severity, particularly in the late-onset phenotype [22]. The randomized controlled study of obese patients with asthma has revealed that a very low-energy diet for 8 weeks led to a mean weight reduction of 14.5% in relation to initial body mass and resulted in improvements in lung function (FEV_1_, FVC), asthma symptoms, and health status [77]. Another study compared the effects of a 10-week diet, physical activity, and combined intervention (caloric restriction + exercise) on asthma in overweight and obese adults. It has been shown that a reduction in body weight by even 5–10% significantly improved asthma control and asthma-related quality of life [78]. In the case of morbid obesity (BMI > 40), an effective option may be bariatric surgery, which brings dramatic weight loss and has a beneficial impact on coexisting asthma. A study by Boulet et al. has demonstrated considerable improvements in airway responsiveness and lung volumes as well as a decrease in the use of asthma treatment after surgically induced weight reduction [79]. Similarly, Dixon et al. have described increased asthma control and quality of life based on their prospective study of obese asthmatics who have undergone bariatric surgery [80].

It should be noted that obesity may change the response to asthma medications and reduce the effectiveness of pharmacotherapy. Peters-Golden has conducted a post hoc analysis of four studies with more than 3000 asthmatics classified by BMI and receiving one of the following treatments: inhaled corticosteroid (beclomethasone), leukotriene receptor antagonist (montelukast), or placebo. The primary endpoint was asthma control days, and, for this parameter, an increase in BMI was associated with a decrease in response to beclomethasone. However, the response to montelukast was not significantly affected [81]. Another study has shown that the odds of achieving asthma control using the inhaled corticosteroid fluticasone or the combination of fluticasone and the long-acting β2-agonist salmeterol in obese patients are markedly lower compared to nonobese asthmatics [82]. It may be due to the fact that obese asthmatics often have predominantly neutrophilic, not eosinophilic, airway inflammation, which is characterized by resistance to inhaled corticosteroid treatment. Impaired drug penetration into the respiratory tract in obese patients may also contribute to this phenomenon [83,84]. The negative impact of obesity on asthma medication activity is also revealed in the case of theophylline. A double-blind study comparing its effect with montelukast and a placebo in asthmatics with different BMI values has shown that in the group treated with theophylline, the risk of asthma exacerbation was considerably higher in obese patients than in overweight or normal-weight patients [38]. A chance for effective treatment could be created by new monoclonal antibodies such as omalizumab, which inhibits the binding of IgE to their high-affinity receptor, or mepolizumab, which acts through interleukin 5 antagonism. However, they show the most effect in asthma with severe eosinophilia, which is not necessarily the case in asthma associated with obesity, especially in the late-onset variant [23]. The administration of omalizumab may also be limited since its dosing is based on the initial IgE level–bodyweight combination and use outside the dosing table is not recommended [21]. Furthermore, recent studies have shown that obesity is one of the factors that reduce the effectiveness of omalizumab in severe asthma [85]. However, more promising results were obtained with the second biological drug mepolizumab. Cluster analysis of clinical data by Ortega et al. has shown that the largest benefit from mepolizumab treatment was achieved in the obesity group, with elevated levels of blood eosinophils and an average duration of asthma of 18 years. The reduction in exacerbation of the disease was much more pronounced in obese asthmatics compared to nonobese subjects, at a level of 67% and 35% respectively [86]. Some authors suggest that this particular group may include patients with the early-onset form of obesity-associated asthma and that the use of mepolizumab in this case could be very effective [20].

## 5. Peptides in Asthma–Obesity Phenotype

Regulatory peptides possess a wide variety of functions acting as hormones, neurotransmitters, or inflammatory mediators participating in the course of asthma, among other diseases [87,88,89,90,91]. Many of them, due to their pro- or anti-inflammatory character, are involved in modulating inflammation, which is the crucial link between bronchial asthma and obesity [92,93,94]. There has been significant progress recently in elucidating the properties of particular peptides, which may shed some light on their role in the mechanisms leading to the development of the asthma–obesity phenotype.

### 5.1. Leptin

Leptin is a 167 amino acids peptide encoded by the obesity (*ob*) gene and secreted primarily by adipocytes [95]. It exerts central action via receptors located in the hypothalamus, regulating energy balance by a reduction of food intake and stimulating energy expenditure [96]. Leptin receptors (Ob-R) exist in at least six isoforms and show significant similarity to the IL-6-type cytokine receptors. They are distributed in the periphery and the lung, kidney, bone marrow, and adrenal gland, as well as on monocytes, neutrophils, and T cells [97,98]. Their presence in the lungs of murine fetuses allows for the assumption that in mice leptin may participate in the process of lung development [99]. In obese patients, serum leptin levels are more than four times higher compared to those of normal weight and correspond strongly with the percentage of body fat [100]. A constantly high concentration of leptin leads to the insensitivity of the hypothalamus to its action and, as a consequence, the development of resistance to leptin and a loss of satiety control function [101]. A study showed that children with a BMI ≥ 85th percentile are characterized by higher leptin resistance compared to those with a lower BMI. However, the leptin resistance in children does not correlate with in utero exposure to maternal overweight/obesity or gestational diabetes mellitus [102]. It has been documented that leptin, in addition to its role in energy regulation, also has proinflammatory properties, affecting various cells involved in the reaction of inflammation. As mentioned above, leptin can stimulate the production of inflammatory mediators such as TNF-α and IL-6 from adipose tissue [44] as well as modulate the lymphocyte response by promoting the Th1 cells pattern, which is reflected in an increase in the interferon gamma (IFN-γ) level [103]. In addition, leptin can enhance the generation of reactive oxygen species in neutrophils [104]. All of these mediators are identified as playing an important role in the pathophysiology of asthma, and, in fact, it has been demonstrated that an increasing leptin concentration is correlated with asthma exacerbation [105] and asthma severity [106]. Some authors put forward a hypothesis that in obesity leptin resistance may reduce the level of α-1 antitrypsin, which, in turn, enhances neutrophil elastase (NE) action, and NE overactivity results in the degradation of lung-tissue proteins, contributing to the development of pulmonary-related diseases including asthma and chronic obstructive pulmonary disorder [107]. The serum concentration of leptin was also shown to be significantly higher in obese asthmatics in comparison to nonobese patients with asthma [108,109], while another study showed that there is no independent association between asthma and levels of leptin, adiponectin, or other markers related to obesity [110]. However, the results of a recent systematic review and meta-analysis from 2022, including fifteen independent studies, support the significant role of leptin in the pathogenesis of asthma in obese individuals by activating inflammatory pathways and the parasympathetic system, which can lead to constriction of the airway and bronchial hyperresponsiveness [111].

### 5.2. Adiponectin

Adiponectin, which is produced and released in large amounts by adipose tissue, plays an important role in energy homeostasis and insulin sensitivity [112]. It consists of 244 amino acids and is synthetized as a monomer, which then combines into trimeric, hexameric, and multimeric structures, forming low, medium, and high molecular weight molecules (LMW, MMW, and HMW, respectively), wherein the HMW isoform is considered to be the most biologically active [113,114]. Adiponectin acts through four currently identified receptors, including adiponectin receptor 1 (AdipoR1), adiponectin receptor 2 (AdipoR2), T-cadherin, and calreticulin [115]. It has been demonstrated that they are present on airway smooth muscle cells [116], lung epithelial cells [117], and pulmonary vasculature [118]. Adiponectin may also contribute to inflammatory processes and is described as a predominantly anti-inflammatory peptide, as it stimulates the production of beneficial mediators, such as IL-10 and IL-1 receptor antagonists [119], as well as inhibits the proinflammatory cytokines IL-6 and TNF-α and nuclear factor-kappa B (NF-κB) signaling [120,121]. However, there are reports indicating that, under certain conditions, adiponectin can have proinflammatory properties; for example, it is involved in the pathogenesis of inflammatory joint diseases such as arthritis [122]. In obesity, the plasma concentration of adiponectin is significantly decreased [123], and, therefore, its limited anti-inflammatory activity may be responsible for the higher risk of inflammatory diseases such as asthma in obese individuals [124]. Although some studies suggest a lack of association between adiponectin and asthma or lung function [125], several authors have found such a correlation, both in animal models and human studies. Adiponectin has been shown to reduce response to methacholine and airway inflammation induced by allergen exposure in mice [126]. Furthermore, patients suffering from severe asthma were characterized by lower adiponectin compared to a mild-to-moderate form. During asthma exacerbation, the serum level of adiponectin was markedly declined, whereas the leptin–adiponectin ratio was significantly elevated [127]. Similar results were obtained in studies on the co-occurrence of asthma and obesity since obese asthmatics have a significantly lower level of adiponectin compared to healthy controls [128] or nonobese patients with asthma [129,130]. In the murine model of obesity-related asthma, exogenous administration of adiponectin led to a reduction in airway resistance, oxidative stress, and inflammatory reaction in lung tissue, confirming that adiponectin contributes substantially to the asthma–obesity phenotype [115].

### 5.3. Resistin

Resistin was first described in rodents as an adipokine that can resist insulin activity [131]. It is a member of the resistin-like molecules (RELMs), also named FIZZ ‘found in inflammatory zone’ family [132]. Human resistin, which is composed of 108 amino acids, forms biologically active oligomers and is expressed mostly in bone marrow, macrophages, mononuclear leucocytes, and, to a lesser extent, adipocytes. The opposite is in the case of mice, where adipose tissue is the major source of resistin [133,134]. In addition, it has been shown that the serum level of resistin is elevated in obesity induced by a high-fat diet and in murine genetic models of disease [131]. Resistin is also involved in the development of inflammation and acts as a proinflammatory agent; it induces NF-κB activity in peripheral blood mononuclear cells [135], increases the expression of TNF-α, IL-6, and IL-8 in white adipose tissue [136], and induces secretion of TNF-α and IL-12 in macrophages [137]. The presence of resistin has been documented in the respiratory tract [138]. However, specific receptors for resistin have not yet been determined, and its actual role in asthma remains inconclusive. In the study of children with asthma, the level of resistin was lower in atopic asthmatics compared to nonatopic asthmatics and healthy controls. There was also a positive correlation between resistin and methacholine PC_20_ and a negative correlation with serum total IgE or eosinophil count [132]. On the other hand, in a cohort of adult patients with asthma, the level of resistin was significantly higher compared to a control group and increased along with the severity of the disease [139]. More consistent results have been obtained in studies on obesity-related asthma. In analysis of human lymphocytes from asthmatic patients, resistin production was strongly enhanced in obese subjects with intermittent or severe persistent asthma [140]. These observations are confirmed by other authors who showed that resistin and the resistin–adiponectin ratio were higher in asthmatics and a more severe state of the disease. Furthermore, the resistin–adiponectin ratio reached the highest value in the group of obese men with asthma [141]. However, the results of other studies led to the conclusion that the serum resistin level is higher in asthmatics, regardless of comorbid obesity [142].

### 5.4. Omentin

Omentin is a novel 313 amino acids adipokine, previously known as intelectin, which was originally identified in intestinal cells, endothelial cells, and then in omental adipocytes [143,144]. It exists in two homologous isoforms. However, in human plasma, the main circulating form is omentin-1 [145], and, so far, it has been better described than omentin-2. One of the biological activities of omentin is to enhance insulin sensitivity in adipose tissue and, thus, contribute to the pathogenesis of obesity [146]. It has been demonstrated that lean patients are characterized by a markedly higher plasma concentration of omentin-1 compared to overweight and obese individuals. Furthermore, omentin-1 levels were negatively correlated with BMI, insulin resistance, or leptin concentration and positively associated with adiponectin and HDL cholesterol levels [145]. In fact, many authors point to the protective role of omentin in various diseases through its anti-inflammatory effect by inhibiting TNF-α activity or the NF-κB signaling pathway [143]. However, its role in bronchial asthma seems to be controversial, and further studies are not facilitated by the fact that there is currently no defined receptor for omentin, although its presence has been confirmed in the lungs [146]. Measurements in the sputum of asthma patients indicated a significantly higher level of omentin-1 during acute exacerbations compared to stable asthmatics or healthy controls, and this increased concentration was related to a subgroup of subjects with elevated levels of eosinophils. In addition, omentin-1 has been shown to be an important component of pathological mucus in acute severe and fatal asthma [147]. In another study, administration of IL-13 led to elevated omentin expression in mouse lungs and in normal human bronchial epithelial cells [148]. Omentin also turned out to be necessary for the IL-13-induced production of monocyte chemotactic protein (MCP)-1 and -3 in murine lung epithelial cells, and, therefore, it can be involved in allergic airway inflammation [149]. Recent research has revealed that omentin expression in asthmatic bronchial epithelial cells is associated with Th2-related parameters, e.g., FeNO and IgE levels [150]. Furthermore, it promotes allergen-induced upregulation of thymic stromal lymphopoietin (TSLP), IL-25, and IL-33 and type-2 reaction in murine models of allergic asthma [151]. However, different results were obtained in a study showing that the plasma level of omentin was significantly lower in severe persistent asthmatics compared to a healthy control, and it inversely correlated with the percentage of Th17 and Th9 cells, which participate in the allergic inflammatory response [152]. Although it was demonstrated that serum omentin level negatively correlates with BMI in asthmatic men [153], understanding the clear impact of omentin on asthma or the co-occurrence of asthma and obesity requires further analysis.

### 5.5. Chemerin

Chemerin is an adipose-derived peptide, synthesized as a 163 amino acid pre-pro-chemerin, which is transformed and secreted in the form of 143-residue pro-hormone. Chemerin biological activity is achieved after proteolytic detachment of the C-terminus fragment of polypeptide [154]. Interestingly, this final modification is responsible for the properties of the molecule, since proinflammatory activity requires processing by serine proteases and the inhibitory effect is a result of processing by cysteine ones [155]. Chemerin is a natural ligand for the chemokine-like receptor 1 (also known as CMKLR1 or ChemR23), which is distributed in adipocytes, inflammatory cells, heart, bone, or lungs. However, two new receptors for chemerin, chemokine receptor-like 2 (CCRL-2) and G protein-coupled receptor-1 (GPR-1), have recently been identified [156]. Chemerin is considered an important chemoattractant in immune reactions and affects functions related to adipose tissue, lipid metabolism, and adipogenesis [157]. In obesity, the levels of chemerin and its receptor CMKLR1 were significantly enhanced, which was associated with inflammation, and TNF-α treatment markedly elevated the chemerin mRNA levels in visceral adipocytes of obese subjects [158]. In fact, serum chemerin concentration was demonstrated to be strongly correlated with biomarkers such as CRP, IL-6, or TNF-α [159], and it substantially increases in various inflammatory diseases [156]. In persistent severe asthma, the level of chemerin, contrary to omentin, was significantly higher compared to control group and displayed a positive correlation with the percentages of Th17 and Th9 cells [152]. On the other hand, exogenous chemerin attenuated allergic inflammation and airway hyperresponsiveness in the murine model of ovalbumin (OVA)-induced asthma by suppressing CCL2 production and inflammatory dendritic cell recruitment [160]. Moreover, chemerin CMKLR1 receptor agonist reduced allergic airway response in mice, which was manifested in the diminished production of epithelial mucus, decreased leucocyte infiltration, and lower number of reactive bronchial epithelial cells in histological analysis, as well as a reduction of the eosinophils count in bronchoalveolar (BAL) fluid [154]. However, in mice with obesity induced by genetic deficiency in carboxypeptidase E (Cpe^fat^ model), sensitized and challenged with OVA, levels of chemerin in BAL fluid and other markers, including IL-4 or IL-13, were significantly higher compared to wild-type counterparts [161]. It could suggest a proinflammatory effect of chemerin in the obesity-related asthma phenotype; nevertheless, further research is necessary.

### 5.6. Visfatin

Visfatin belongs to the adipokines family, and it is also known as nicotinamide phosphoribosyltransferase NAMPT or pre-B cell colony enhancing factor PBEF [162]. This 491 amino acids peptide is expressed in different tissues, including the heart, liver, spleen, and lung, as well as leukocytes and visceral adipose tissue, hence the term visfatin [162,163]. It binds to the insulin receptor (IR), reduces the release of glucose from the liver, increases its utilization by muscle and adipocyte cells, and, as a consequence, leads to a decrease in blood glucose levels [163]. However, despite a beneficial insulin-mimicking effect, visfatin is considered a proinflammatory agent and is markedly upregulated in various inflammatory conditions [164]. Visfatin has been shown to stimulate the expression of inflammatory mediators, such as IL-1β, IL-6, or TNF-α [165], promote chemotaxis in B cells and monocytes [166], and inhibit neutrophil apoptosis [167]. The relation between visfatin and obesity has not yet been clearly defined. In the majority of studies, the level of circulating visfatin is significantly increased in obese patients compared to a control group [168,169,170]; however, other authors point to the lack of such a relationship or even a negative correlation between visfatin and BMI [171,172]. In the case of asthma research, the results also remain conflicting. The serum concentration of visfatin was shown to be considerably higher in asthmatics than in the healthy group [173], and visfatin stimulates the expression of mucins MUC8 and MUC5B, the main components of mucus, in human airway epithelial cells [174]. However, some studies indicated the opposite relation, since visfatin levels were markedly lower in obese and nonobese children with atopic asthma compared to healthy controls, and there was no association between visfatin and BMI, spirometric parameters, severity of asthma, or degree of allergic sensitization [175]. Nevertheless, several studies in rat models of asthma and obesity have suggested an unfavorable role of visfatin in the co-occurrence of both diseases. The animals with diet-induced obesity and OVA sensitization were characterized by significantly increased expression of visfatin mRNA in trachea tissue in comparison to the normal diet, normal diet OVA-sensitized, and high-fat diet groups [176]. In a similar experimental model, both asthma and obesity stimulated the pathological changes in the lungs, which were accompanied by enhanced visfatin mRNA and NF-κB protein levels in the airway tissue [177]. Another study is consistent with these observations since hyperresponsiveness to methacholine and elevated visfatin levels in the trachea were demonstrated in ovalbumin-sensitized obese rats [178].

### 5.7. Ghrelin

Ghrelin, unlike the previously mentioned adipokines, is secreted mainly by the stomach and to a lesser degree by the jejunum, duodenum, urogenital organs, pituitary, and lungs [179]. The peptide consists of 28 amino acids and is described as the endogenous ligand of the growth hormone secretagogue receptor (GHSR) 1a, which promotes the release of growth factor from the anterior pituitary gland [180]. It exhibits a wide range of different functions, both at the central and peripheral levels. Ghrelin is well-known for its orexigenic properties since it stimulates appetite and increases food consumption by activating its receptors in the hypothalamic neurons [179,180]. Many authors have reported that the concentration of circulating ghrelin in obesity is significantly lower compared to lean subjects [181,182]. This may result from a high caloric intake by obese patients, and the reduction of body mass leads to an increase in ghrelin levels [183]. Ghrelin can modulate the response to inflammation and is perceived as an anti-inflammatory peptide, inhibiting the expression of proinflammatory mediators, such as TNF-α, IL-1β, or IL-6 [184]. A negative correlation between plasma ghrelin concentration and IgE level was also observed [185]. Despite the fact that ghrelin is expressed in the human lungs [186] and its receptor binding sites have been identified in the lung parenchyma and pulmonary artery wall [187], the exact role of ghrelin in bronchial asthma is still under investigation. In part of the studies, serum ghrelin levels were significantly elevated in asthmatic subjects compared to patients without asthma [173,188] and, again, higher in uncontrolled asthma than in a controlled state [188]. However, according to the authors, this indicates the anti-inflammatory effect of ghrelin and results from a compensatory mechanism of counteracting the proinflammatory cytokines’ activity [173,188]. In other studies, there was no correlation between serum ghrelin level and atopy, asthma, or lung function [125], nor between ghrelin concentration in children with or without a previous diagnosis of asthma [189]. However, stable asthma was characterized by lower ghrelin compared to a control group, and during asthma exacerbation, the level of ghrelin was markedly decreased [127]. Furthermore, in the murine model of OVA-induced asthma, ghrelin alleviated unfavorable changes in the course of inflammation, including pathologic features in the histological picture, airway hyperresponsiveness, increased number of total and particular leucocytes, and elevated levels of TNF-α, IFN-γ, IL-5, or IL-13 [190]. The research conducted on a group of children with asthma and obesity showed that serum ghrelin concentration was decreased in obese asthmatics compared to nonobese asthmatic subjects as well as was lower in nonobese asthmatics than in healthy controls. These results may suggest the anti-inflammatory effect of ghrelin in the pathogenesis of obesity-associated asthma [130].

### 5.8. Glucagon-like Peptide 1

Glucagon-like peptide 1 (GLP-1) is a 30–31 amino acids incretin hormone produced by intestinal L-cells and neurons in the nucleus of the solitary tract in the brainstem in response to nutrient intake [191,192]. Its main function is to increase insulin secretion in pancreatic β cells as well as reduce glucagon release and slow stomach emptying [192]. GLP-1 acts through the activation of G-protein-coupled receptors. GLP-1R and GLP-1R agonists are already approved for type-2 diabetes mellitus (T2DM) and obesity treatment [193]. The early research has demonstrated that the level of plasma GLP-1 after oral carbohydrate ingestion is considerably decreased in obese patients in comparison to a lean group [194]. GLP-1, in addition to participating in glycemic control, has anti-inflammatory properties under many different conditions [195]. Given the fact that GLP-1R receptors are present in the respiratory tract, including airway smooth muscle cells, pulmonary vasculature, or type-II alveolar cells [193], GLP-1 may play an important role in the inflammatory processes associated with asthma. In fact, in a murine model of OVA-induced asthma, GLP-1 analog liraglutide significantly reduced airway inflammation, mucus hypersecretion, and elevated levels of E-selectin or MUC5AC mucin [196], whereas another GLP-1R agonist, exendin-4, showed a bronchorelaxant effect, alleviating hyperresponsiveness of human isolated bronchi subjected to passive sensitization and high-glucose concentration [197]. Furthermore, lipopolysaccharide stimulation of eosinophils collected from allergic asthmatics led to increased production of the proinflammatory cytokines IL-4, IL-8, and IL-13, which was attenuated by GLP-1 analog treatment [198]. Similar positive conclusions come from human research since a small cohort study on patients with T2DM with concomitant asthma showed that 52 weeks of liraglutide therapy resulted in both weight loss and reduction in the number of asthma exacerbations [199]. In another clinical trial, liraglutide exerted a beneficial influence on forced vital capacity in individuals suffering from T2DM [200]. However, a recent network meta-analysis found that GLP-1R agonists did not significantly affect the risk of asthma incidents [201]. In the case of asthma with obesity or metabolic syndrome, the level of GLP-1 decreases, which correlates with more pronounced insulin resistance, reduced NO production, and increased smooth muscle contractility, and, as a consequence, may lead to bronchoconstriction [202]. Dysfunction in arginine metabolism or enhanced formation of advanced glycation end products (AGEs) also seem to be important in obese asthma, and these mechanisms can be modified by GLP-1 [203]. The critical role of GLP-1R agonists has recently been established in murine models of the disease. In mice with obesity induced by a high-fat diet and OVA-induced asthma, liraglutide ameliorated airway eosinophilic inflammation and hyperresponsiveness to methacholine, as well as decreased expression of proinflammatory cytokines IL-4, IL-5, and IL-33, and suppressed activity of NLRP3 inflammasome, caspase-1, and IL-1β in lung tissue. These experiments may reflect an early-onset asthma–obesity phenotype in humans [204]. Liraglutide has also been proven to be effective in obese polygenic mice (TALLYHO model), undergoing challenges with *Alternaria alternata* extract. Administration of a GLP-1 analog decreased level of IL-33 and TSLP in BAL fluid, the number of ILC2 cells in the lung, lung levels of IL-5, IL-13, and eotaxin, hyperresponsiveness to methacholine, and expression of ICAM-1 on airway endothelial and epithelial cells, along with suppression of allergen-induced neutrophilia and production of KC and LIX, chemoattractants for neutrophils [205]. Another substance already used in medicine, dulaglutide, was studied in obesity-induced AHR and lung fibrosis since obesity characterized by weight gain ≥ 145% without any allergen exposure can lead to the development of non-Th2 type/noneosinophilic asthma. Dulaglutide demonstrated a beneficial effect on airway hyperresponsiveness, the number of inflammatory cells in BAL fluid, mostly neutrophils and macrophages, and the levels of IL-17, TGF-β, and IL-1β in lung homogenates, as well as alleviated the fibrosis and differentiation of Th1 and Th17 cells [206]. These findings suggest that GLP-1 analogs could significantly contribute to the therapy of obesity-related asthma.

### 5.9. Cholecystokinin

Cholecystokinin (CCK) is a well-known satiety hormone, which inhibits food intake in response to lipids and proteins as well as increases gallbladder contraction, secretion of pancreatic enzymes, and delays gastric emptying [207]. It is released by enteroendocrine cells in the mucosa of the small intestine and specialized neurons in the nervous system. The precursor of the hormone is pre-pro-CCK, a polypeptide containing 115 amino acids, which is further transformed into shorter, biologically active forms, such as CCK-58, CCK-39, CCK-33, CCK-22, or CCK-8 [208]. To date, two cholecystokinin receptors have been identified, due to anatomical location previously referred to as CCK-A ‘alimentary type’ and CCK-B ‘brain type’, and now described as CCK-1 and CCK-2, respectively [209]. Several studies have demonstrated that CCK-8 modulates functions of inflammatory cells, such as lymphocytes or neutrophils [210,211], and affects cytokine secretion and NF-κB activation with a predominant anti-inflammatory activity [212,213]. On the contrary, there is also research showing that reduction in the chronic inflammatory state was achieved through the antagonism of the CCK receptor [214]. In obesity, the satiety effect of CCK seems to be weakened [207], and its plasma level is significantly increased, both in obese patients [215] and in animal models of disease [216]. The studies on the impact of CCK on asthma are, in turn, very limited. It has been shown that CCK-8 induced a concentration-dependent constriction in a guinea pig trachea, as well as in large human airways [217]. A recent study has also revealed that CCK-1 receptors are widely expressed in human airway smooth muscle (ASM), and administration of a CCK agonist led to a contractile response of ASM cells. Furthermore, mice with a high-fat diet or genetically induced obesity were characterized by an increased level of CCK in the lungs and the administration of the CCK-1 antagonists suppressed the innate airway hyperresponsiveness to methacholine in obese animals. These results suggest that blocking of CCK receptors may have a beneficial effect in the case of co-occurring asthma and obesity [218].

### 5.10. Substance P

Substance P (SP), a member of the tachykinin family of neuropeptides, is released from central and peripheral endings of primary afferent neurons, acting as a neurotransmitter [219], but is also produced in many different non-neuronal cells, particularly in the inflammatory state [220]. This 11 amino acids molecule is involved in a wide variety of physiological and pathological processes, including nociception and neurogenic inflammation [221]. Tachykinins exert their functions through three specific receptors NK, and although SP is able to stimulate all of them, it preferentially binds to the NK1 receptor [222]. It has been demonstrated that SP is present in airway smooth muscle cells, airway epithelial cells, and cells participating in an inflammatory response, such as lymphocytes, monocytes, eosinophils, and macrophages [220]. Activation of the NK1 receptor in airways leads to mucus secretion, vasodilatation, microvascular leakage, and infiltration of inflammatory cells, whereas stimulation of the NK2 receptor may cause bronchoconstriction, due to the presence of NK2 on smooth muscle [223]. In asthma, the concentration of SP both in the BAL fluid and the induced sputum increases compared to healthy controls [224,225]; nevertheless, no significant difference in lung parenchymal level of SP between asthmatic and nonasthmatic patients was also observed [226]. Another in vivo study revealed that SP inhalation in asthmatics results in acute airway narrowing in a dose-dependent manner [227]. Furthermore, a systematic review of seven trials, showed that NK receptor antagonists can improve lung function and reduce airway responsiveness, although the influence on airway inflammation or asthma symptoms was poorly or not defined [228]. However, the role of SP as a proinflammatory agent is well described since it induces the NF-κB pathway and enhances proinflammatory cytokines secretion, including IL-1, IL-6, IFN-γ, or TNF-α [229]. In the respiratory tract, SP is released from the nonadrenergic, noncholinergic nerve system (NANC) in response to thermal, mechanical, chemical, or inflammatory stimuli [228], and as part of the neurogenic inflammatory mechanism, it exerts tissue-specific reactions [222]. In obesity, the plasma content of SP is significantly increased compared to the control group [230] and SP is positively correlated with the BMI value [231]. Furthermore, the administration of the SP antagonist in diet-induced obese and genetically obese mice led to a reduction in weight and adiposity, decreased appetite, and improved response to insulin action [232]. A study on obesity and ovalbumin asthma in mice has shown elevated expression of NK1 receptors on lung epithelium and adipose tissue, suggesting that both of these anatomical areas may be affected directly by SP [229]. In another experiment, the association between asthma, obesity, and SP has been confirmed, and the SP level positively correlated with glycemia, number of eosinophils and mast cells in BAL fluid, peribronchial inflammatory reaction, and concentration of OVA-specific IgE. Furthermore, neurogenic inflammation seems to be an important mechanism in the asthma–obesity phenotype in a murine model, and although these two conditions independently enhanced the serum level of SP, the highest concentration was achieved in the coexistence of both diseases [233]. In further studies, the impact of NK1 antagonist application has been examined. Blockade of the NK1 receptor in obese asthmatic mice resulted in significant weight loss, decreased food and energy intake, lowered adipocyte areas, reduced levels of serum parameters, such as glucose, insulin, resistin, IL-6, and anti-OVA IgE, and ameliorated peribronchial inflammation, as well as lower number of eosinophils in BAL fluid in comparison to nontreated animals. All these results support the idea that SP may be a promising therapeutic target for asthma in the context of obesity [234].

### 5.11. Neuropeptide Y

Neuropeptide Y (NPY), together with pancreatic polypeptide (PP) and peptide YY (PYY), belongs to the peptide family that acts through five subtypes of G-protein-coupled receptors described as Y1, Y2, Y4, Y5, and Y6 [235]. NPY consists of 36 amino acids and is widely distributed in many regions of the brain and on the periphery, including sympathetic nerves, where it is colocalized with catecholamines [235,236] and becomes a major hormone that is increased during chronic stress [237]. NPY is considered a specific link between the nervous and immune systems since Y1 receptors are expressed on different inflammatory cells such as macrophages, lymphocytes, mast cells, or dendritic cells [236]. It exerts a modulatory impact on Th cell differentiation, natural killer cell activation, or release of inflammatory mediators [238]. However, the exact role of NPY in inflammation seems to be ambiguous, and some studies indicate a bimodal character of this peptide in the immune reaction with an opposing effect on T cells and antigen-presenting cells [239]. At the same time, NPY is strongly involved in appetite regulation, its central administration leads to higher food intake and weight gain, and chronic treatment can result in the development of obesity [240]. NPY is crucial for diet- or stress-induced fat-mass accumulation, and its overexpression in noradrenergic neurons altered glucose tolerance as well as increased adiposity in the transgenic mouse model [241]. Nevertheless, in another study, Y1 receptor-deficient mice unexpectedly exhibited moderate obesity and modest hyperinsulinemia, which can suggest that the Y1-R deficiency is compensated by other receptors responsible for NPY signaling, e.g., Y5-R [242]. NPY is present in the respiratory tract, in the nerves that innervate the smooth muscle of the bronchi, glands, submucosa, and vasculature of the airways [89], pointing to its significant contribution to the pathophysiology of asthma. Indeed, it has been demonstrated that NPY induced a constricting effect on trachea, bronchi, and lung parenchyma preparations collected from guinea pigs [243]. NPY receptors were also shown to be constitutively expressed in structural and inflammatory cells in murine lungs [244]. Furthermore, the serum concentration of NPY is elevated during asthma exacerbation [238], and in the group of elderly asthmatic patients, the level of NPY was significantly higher both in the stable resting state and in acute severe condition compared to control subjects [245]. However, other authors reported that NPY concentration was not markedly different between children with and without asthma [246] or was even lower in young adult asthmatics compared to healthy controls [247]. In animal studies, NPY aggravated allergic airway inflammation through activating the Y1 receptor in an ovalbumin model, and NPY- or Y1-knockout mice were characterized by a significantly reduced number of eosinophils in the bronchoalveolar fluid, lower serum IgE concentration, and attenuation of the Th2 immune response [248]. Similar results were obtained from the experiment using house dust mites for sensitization and airway challenge, showing that, in NPY-deficient mice, hyperresponsiveness to methacholine, eosinophil count, and level of Th2 cytokines (IL-4, IL-5, and IL-13) in the BAL fluid were significantly decreased in comparison to the control group. Similarly, the administration of an NPY antagonist to wild-type animals markedly suppressed the AHR and inflammatory state in murine airways [249]. To date, the number of reports on the correlation between asthma, obesity, and NPY is very limited. In a clinical study of asthma among young adults, there was no evidence suggesting a significant interactive impact of NPY level and obesity on the increased prevalence of asthma [250]. However, another study revealed that overweight is related to a 2.5-fold increased risk of asthma in patients with particular alleles in their *NPY* genotype [251].

## 6. Perspectives and Conclusions

Despite the high global prevalence of obesity and bronchial asthma and the increasing co-occurrence of both diseases, there are currently no specific recommendations for the treatment of patients suffering from these conditions. A very important intervention, the effectiveness of which has been confirmed in several studies, is weight loss [77,78]. It can be recommended, preferably under the supervision of a dietitian and nutritionists, for almost every obese asthmatic. Particular attention should be paid to the problem of childhood asthma with obesity [252], which may have intensified due to increasing weight gain during the recent pandemic, caused by less physical activity and modified eating habits in children and their families [253]. Therapies related to the gut microbiome, including fecal microbial transplantation, probiotics, prebiotics such as dietary fiber, and rational use of antibiotics can also be considered [254]. However, there is still an urgent need to search for new therapeutic solutions, and regulatory peptides, which affect a number of mechanisms involved in the development of asthma and obesity, could play a key role in this area. Current knowledge of peptides, such as adiponectin or glucagon-like peptide 1, allows one to assume that they have a beneficial influence on the asthma–obesity phenotype, and, in fact, GLP-1 agonists are already used in the treatment of obesity. Ghrelin also appears to have a positive effect, but it requires more extensive investigation in this field. On the contrary, an adverse effect on obese asthma has been confirmed in the case of leptin, cholecystokinin, or substance P, however, application of receptor antagonists for these peptides may provide substantial therapeutic benefits. The role of resistin and neuropeptide Y, similar to ghrelin, is not yet sufficiently understood, but it appears that they have a rather negative impact on asthma with obesity. Moreover, the inconclusive results obtained in studies on new adipokines, including omentin, chemerin, and visfatin, indicate the need for a more detailed analysis of their contribution to asthma alone and obesity-related asthma. The results of studies on particular peptides in the asthma–obesity phenotype are summarized in Table 1.

It is also worth noting that the peptides described above exert their action in a complex environment, and some of them may interact and influence each other. For example, the plasma level of omentin in humans has been shown to be inversely correlated with leptin and positively correlated with adiponectin concentrations [145]. Similarly, in mouse models of asthma with obesity, administration of GLP-1 analog increases the level of adiponectin and decreases the level of leptin [204], whereas, in another study, the SP receptor antagonist reduces the serum concentration of resistin [234]. This seems to be an important issue in the context of the therapeutic effectiveness of potential medications. In conclusion, scientific interest in peptides remains strong; however, further research is necessary to understand their true role in the asthma–obesity phenotype. It is conceivable that from a further perspective, they could become promising therapeutic targets, creating opportunities for more effective therapy.

## Figures and Tables

**Table 1:** Results of studies investigating selected peptides in asthma–obesity phenotype.

Peptide	Type of Study	Main Results	Reference
Leptin	Human Study	Serum leptin level in obese asthmatics is significantly higher compared to nonobese asthmatics	[108]
	Human Study	Serum leptin level in obese asthmatics is significantly higher compared to nonobese asthmatics	[109]
	Prospective Cohort Study	Asthma is associated with obesity in adults, but results of study do not support a significant role of leptin in this correlation	[110]
	Systematic Review and Meta-analysis	Leptin plays an important role in the pathogenesis of asthma in obese individuals	[111]
Adiponectin	Human Study	Serum adiponectin level in obese asthmatics is significantly lower compared to healthy controls	[128]
	Human Study	Serum adiponectin level in obese asthmatics is significantly lower compared to nonobese asthmatics	[130]
	Prospective Cohort Study	Serum adiponectin level in obese asthmatics is significantly lower compared to nonobese asthmatics	[129]
	Animal Study	Adiponectin ameliorates AHR, airway inflammation, and oxidative stress in murine model of obesity-related asthma	[115]
Resistin	Human Study	Resistin production is strongly enhanced in cell-culture supernatants from obese subjects with intermittent or severe persistent asthma	[140]
	Cross-sectional Observational Study	Plasma resistin level is higher in asthmatics compared to controls, higher in more severe than a mild-to-moderate state, and resistin–adiponectin ratio is highest in obese men with asthma	[141]
	Human Study	Serum resistin level is higher in asthmatics, independent of obesity	[142]
Omentin	Human Study	Serum omentin level is negatively correlated with BMI in asthmatic men	[153]
Chemerin	Animal Study	Chemerin level in BAL fluid is higher in obese than in nonobese mice in OVA-induced asthma model	[161]
Visfatin	Human Study	Serum visfatin level is significantly lower in obese and nonobese children with atopic asthma compared to healthy controls, and there is no association between visfatin and BMI, spirometric parameters, asthma severity, or allergic sensitization degree	[175]
	Animal Study	Visfatin mRNA expression in the trachea tissue is significantly increased in OVA-sensitized obese rats	[176]
	Animal Study	Visfatin level in the trachea tissue is significantly increased in OVA-sensitized obese rats	[178]
	Animal Study	Visfatin mRNA expression in the airway tissue is significantly increased in rat model of asthma and obesity	[177]
Ghrelin	Human Study	Serum ghrelin level is significantly decreased in obese asthmatic compared to nonobese asthmatic children and lower in nonobese asthmatics than in healthy controls	[130]
Glucagon-like peptide 1	Review Article	Asthma with obesity and metabolic syndrome is characterized by decreased GLP-1 level, increased insulin resistance, reduced NO production, and increased smooth muscle contractility, which leads to bronchoconstriction	[202]
	Review Article	GLP-1 can modulate alterations in arginine metabolism or enhanced formation of advanced glycation end products, important mechanisms involved in obese asthma	[203]
	Animal Study	GLP-1 agonist decreases airway eosinophilic inflammation, AHR to methacholine, expression of proinflammatory cytokines, and inflammatory reaction in lung tissue in obese mice with OVA-induced asthma	[204]
	Animal Study	GLP-1 agonist decreases levels of proinflammatory cytokines, number of lung ILC2 cells, AHR to methacholine, and allergen-induced neutrophilia in obese TALLYHO mice challenged with *Alternaria alternata* extract	[205]
	Animal Study	GLP-1 agonist decreases AHR to methacholine, number of inflammatory cells in BAL fluid, levels of proinflammatory cytokines in the lung, and process of fibrosis in murine model of obesity-induced asthma	[206]
Cholecystokinin	Animal Study	Lung CCK level is significantly increased in obese mice and CCK-1 receptor antagonists suppress the innate AHR to methacholine in obese animals	[218]
Substance P	Animal Study	NK1 receptor expression is increased on lung epithelium and adipose tissue in murine obese–asthma model	[229]
	Animal Study	Serum SP level positively correlated with glycemia, peribronchial inflammation, OVA-specific IgE, number of eosinophils and mast cells in BAL fluid, and neurogenic inflammation may be involved in the asthma–obesity phenotype in murine model	[233]
	Animal Study	NK1 receptor antagonist decreases weight, food and energy intake, adipocyte areas, levels of glucose, insulin, resistin, IL-6, OVA-specific IgE, number of eosinophils in BAL fluid, and peribronchial inflammation in obese asthmatic mice	[234]
Neuropeptide Y	Cross-sectional Study	No evidence for significant interactive impacts of NPY level and obesity on the increased asthma prevalence in young adults	[250]
	Human Study	Overweight is associated with 2.5-fold increased risk of asthma in patients with particular alleles in their *NPY* genotype	[251]
